# Auditory Hallucinations in a Deaf Patient: A Case Report

**DOI:** 10.1155/2013/659698

**Published:** 2013-07-09

**Authors:** Natalia Pedersen, René Ernst Nielsen

**Affiliations:** ^1^Team for Psychotic Disorders, Aalborg Psychiatric Hospital, Aalborg University Hospital, Brandevej 5, 9000 Aalborg, Denmark; ^2^Unit for Psychiatric Research, Aalborg Psychiatric Hospital, Aalborg University Hospital, Mølleparkvej 10, 9000 Aalborg, Denmark; ^3^Institue of Clinical Medicine, Aarhus University, 8200 Aarhus, Denmark

## Abstract

This case report describes the progression of symptoms in a young deaf female. Her initial psychotic symptoms occur at the age of 16, but she did not come into contact with a psychiatric treatment facility before the age of 27, where she felt symptoms were distressing. The case report describes the difficulties in evaluating psychotic symptoms in a deaf patient, as well as the use of specialized scales in combination with the standard psychiatric evaluation. The current evidence, concerning the prevalence of psychotic symptoms, as well as the influence of deafness on the understanding of psychosis, is described.

## 1. Introduction

In a review by Landsberger and Diaz [1], they examined the diagnostic and clinical features of deaf psychiatric inpatients and found studies showing prevalence rates of psychotic disorders from 20% to 54%. Authors of early studies suggested that these prevalence rates could represent diagnostic inaccuracies resulting from cultural and linguistic biases [2]. The deaf and hard of hearing population have a “deaf culture” that needs to be considered in the diagnostic procedure; this is especially important in deaf persons who are not fluent in the spoken/verbal language [3]. Due to suboptimal communication between deaf patients and early caregivers and peers, for example, parents and children of the same age, it is reasonable to suspect that the deaf patients could have difficulties with the normal experience of socialization, problem-solving skills, and emotional regulation [4]. The very early functional skill attainment seen in childhood is lacking in deaf patients, which likely contributes to a high rate of deaf patients displaying symptoms of socially inappropriate behaviour, poor self-care, behavioural impulsivity, aggression and self-injury, leading to diagnosis of impulse control disorders, mental retardation, and pervasive developmental disorders [4].

In a recent review, the prevalence rate of psychotic disorders in a sample of deaf inpatients was 43% in the USA [3]. Similar results were found in a population of deaf inpatients living in the UK, with a prevalence rate of 39% [5]. In contrast, studies from other European countries have reported lower prevalence rates with deaf Dutch inpatients having a prevalence rate of eight percent [6] and deaf Austrian inpatients having a prevalence rate of four percent [7].

In this paper, we wish to describe a patient with symptoms of psychosis and congenital deafness and the difficulties observed in our diagnostic process.

## 2. Case Presentation

A 28-year-old single female with family history of depression, was admitted to the psychiatric hospital due to suspicion of auditory hallucinations, with voices encouraging her to do self-harm acts. The patient has a congenital hearing impairment that was diagnosed at the age of two. The patient began learning sign language at the age of ten, and had previously only communicated by lip reading and talking. From the age of ten to 16, the patient received schooling in sign language techniques in a specialized institution for deaf children in Aalborg. Currently, the patient speaks understandable Danish; she still reads lip, but adequate two-way communication is dependent on sign language. 

The patient was interviewed using the Danish version of the Present State Examination (PSE) [8]. Both the patient and the examiner received a transcript of the PSE questions, thereby allowing the patient to read the questions for herself, while they were read out loud by the examiner. Additionally, an interpreter was present to translate each question into sign language for the patient. Due to the general difficulties in the diagnostic process of nonhearing patients, the Atkinson interview was also employed [9]. Ninety-four questions enable the interviewer to identify how much auditory meaning the patient is putting into voice hallucinations how much of it is a communication act with lip reading and sign language, and also if any other types of hallucinations are present. 

### 2.1. Somatic Disease Biography

In October 2009, during a period of two weeks, the patient's full field of vision was blurry on both eyes. Magnetic resonance imaging (MRI) and magnetic resonance angiography (MRA) of the cerebrum were conducted in relation to these symptoms, and the results were normal. In November 2009, Benign intracranial hypertension (BIH) was diagnosed with spinal pressure of 33 mmHg that was accompanied with symptoms of fatigue, headache, and poor concentration. She was treated with acetazolamide, carbonic anhydrase inhibitor, during a three-year period that was phased out without any relapsing symptoms.

The patients hearing difficulties have been investigated on several occasions, last in January 2012, utilizing audiograms, showing that the patient could hear sounds on her left ear; however, she could not recreate any words. On her right ear, she could both hear and successfully recreate words. The right ear was examined with discrimination test, which determines how well the patient can hear sounds and understand speech under amplified volume and by using a hearing apparatus. Thirty-two percent of the time the patient could not distinguish separate words, despite the fact that the sound volume was increased to 85 dB. The conclusion was severe hearing loss.

In November 2012, a computed tomography scan of cerebrum (CTC) was conducted during the psychiatric hospitalization to investigate possible organic aetiologies, and the scan was normal. 

### 2.2. Psychiatric Disorder Biography

The patient presents three episodes of  hallucinatory experiences, as well as symptoms of anxiety, probably related to an assault five years ago.

The initial episode of hallucinations occurred when the patient was 16 or 17 years of age. The hallucinatory voices appeared gradually. Initially, she heard her father's voice, which encouraged the patient with positive comments, similar to his role in her life. Hereafter, she heard her mother's voice, which praised and supported the patient, also in accordance with her real experiences. The voices came from her right ear. Patient describes these voices as being loud in her head, approximately with the same volume as patient's own voice and was perceived quite musically. The volumes of the voices remained constant and had a soothing effect on the patient and were not just hypnopompic or hypnagogic. During the present hospitalization, the patient explains that she has not heard these voices during the last years and that the specific wording of the auditory hallucinations was forgotten. The hallucinations persisted for two to three years and disappeared without pharmacological treatment.

The patient again experienced hallucinatory symptoms, the second time in connection with the death of her cousin, when she was 17 years of age. She saw him as clearly as she could see other people, his face, his clothed body, and smelling his scent. Communication with him was as it used to be when he was alive. The patient describes his voice with an alternating volume, high and low, and she experienced his voice being clear at some time points, but also faint, indistinguishable at other time point, making it difficult to comprehend. She experienced a two-way verbal communication, both by hearing his voice and by lip reading. The auditory hallucination seemed to be external, not originating from inside her head. The patient described nonverbal, or so-called hidden signals, that her diseased cousin sent her; however, she can neither describe the actual way these signals were sent nor their meaning. The patient was aware that other people could not see her dead cousin, but was unable to explain this fact. The hallucinations persisted until pharmacological treatment was initiated during hospitalization.

At the age of  25, the patient was physically assaulted and developed anxiety attacks with autonomic symptoms. The symptoms appeared once or twice a month mostly in the evening when the patient was alone and lasted for approximately 30 minutes. They appeared spontaneously without any triggering factors. They were accompanied by sweating, tachycardia, stomach aches, and flashbacks to the episode of the attack.

The current episode of auditory hallucinations caused her to be admitted to the psychiatric hospital. It occurred in the summer of 2012, where the patient was 27 years old. The patient experienced a male voice that commanded and urged her to perform certain acts, where the patient was supposed to hurt herself or others, for example, “you should take a knife and stab yourself.” The patient characterized the voices as being harmful and threatening. There were no delusions in relation to the auditory hallucinations. The voices were heard in both ears and were of the same volume as the patient's own voice, but they were of a higher pitch and felt loud in her head. The voices appeared intermittently several times a day with duration of five to ten minutes. The symptoms disappeared after initiation of aripiprazole 20 mg per day.

The patient was diagnosed with an ICD-10 F28 diagnosis (other nonorganic psychotic disorder).

## 3. Discussion

The phenomenon of auditory hallucinations among patients with hearing disorders is poorly examined; currently, the nature of this phenomenon is not fully elucidated. In our case, the communication with the patient was compromised, due to the difficulties of understanding questions, which had to be reformulated and repeated. It was challenging for the patient to give comprehensive descriptions of the phenomenon she experienced. 

Bailly et al. [10] find similar difficulties, while working with hearing-impaired children where the assessment of psychiatric disorders sets methodological challenges in relation to verbal communication and diagnostic process. The immature language understanding exhibited by many hearing-impaired or deaf patients hampers accurate psychiatric evaluation. The investigations of psychiatric symptoms are then compromised due to many of the assessment procedures being highly verbal and standardized for normal-hearing. These difficulties may explain that the prevalence rates of mental disorders in hearing-impaired children and adolescents were found to vary from 15% to 60% [10].

The use of PSE may increase the risk of false positive psychotic symptoms, due to the questions being used in a different population, than the originally intended. More specialized scales and questions may improve the diagnostic process, particularly in this subpopulation of patients with hearing impairments, and patients should, in case of any diagnostic ambiguity, be seen by a psychiatrist specialising in patients with hearing difficulties.

## 4. Learning Points

- Auditory hallucinations are more common in patients with hearing difficulties.
- Diagnostic procedures should include specialized scales for the evaluation of psychotic symptoms in patients with hearing deficits.
- A thorough somatic examination including neuroimaging should be conducted if psychotic symptoms are suspected.
